# The determinants of COVID-19 case reporting across Africa

**DOI:** 10.3389/fpubh.2024.1406363

**Published:** 2024-06-27

**Authors:** Qing Han, Ghislain Rutayisire, Maxime Descartes Mbogning Fonkou, Wisdom Stallone Avusuglo, Ali Ahmadi, Ali Asgary, James Orbinski, Jianhong Wu, Jude Dzevela Kong

**Affiliations:** ^1^Africa-Canada Artificial Intelligence and Data Innovation Consortium (ACADIC), Toronto, ON, Canada; ^2^Department of Mathematics and Statistics, York University, Toronto, ON, Canada; ^3^Faculty of Computer Engineering, K. N. Toosi University of Technology, Tehran, Iran; ^4^Disaster and Emergency Management, School of Administrative Studies, York University, Toronto, ON, Canada; ^5^Dahdaleh Institute for Global Health Research, York University, Toronto, ON, Canada; ^6^Artificial Intelligence & Mathematical Modeling Lab (AIMM Lab), Dalla Lana School of Public Health, University of Toronto, Toronto, ON, Canada; ^7^Department of Mathematics, Bahen Centre for Information Technology, University of Toronto, Toronto, ON, Canada; ^8^Global South Artificial Intelligence for Pandemic and Epidemic Preparedness and Response Network (AI4PEP), Toronto, ON, Canada

**Keywords:** case reporting, COVID-19, Africa, determinants of case reporting, generalized additive model, hierarchical clustering on principal component analysis

## Abstract

**Background:**

According to study on the under-estimation of COVID-19 cases in African countries, the average daily case reporting rate was only 5.37% in the initial phase of the outbreak when there was little or no control measures. In this work, we aimed to identify the determinants of the case reporting and classify the African countries using the case reporting rates and the significant determinants.

**Methods:**

We used the COVID-19 daily case reporting rate estimated in the previous paper for 54 African countries as the response variable and 34 variables from demographics, socioeconomic, religion, education, and public health categories as the predictors. We adopted a generalized additive model with cubic spline for continuous predictors and linear relationship for categorical predictors to identify the significant covariates. In addition, we performed Hierarchical Clustering on Principal Components (HCPC) analysis on the reporting rates and significant continuous covariates of all countries.

**Results:**

21 covariates were identified as significantly associated with COVID-19 case detection: total population, urban population, median age, life expectancy, GDP, democracy index, corruption, voice accountability, social media, internet filtering, air transport, human development index, literacy, Islam population, number of physicians, number of nurses, global health security, malaria incidence, diabetes incidence, lower respiratory and cardiovascular diseases prevalence. HCPC resulted in three major clusters for the 54 African countries: northern, southern and central essentially, with the northern having the best early case detection, followed by the southern and the central.

**Conclusion:**

Overall, northern and southern Africa had better early COVID-19 case identification compared to the central. There are a number of demographics, socioeconomic, public health factors that exhibited significant association with the early case detection.

## 1 Introduction

The ongoing COVID-19 pandemic, triggered by the SARS-CoV-2 virus (1), has spread globally with noticeable variations in reported cases and deaths across different regions. As of May 5, 2022, the pandemic is affecting more than 220 countries and territories, with over 513 million cases and 6 million deaths worldwide (2). In particular, the spread of the virus and subsequent reporting of cases have shown a slower pace in African nations. Since the identification of the first case in Egypt on February 14, 2020 (3), the growth of new infections within African countries remained relatively modest, with the WHO African region reporting over 8 million cases and 170,000 deaths (4), a stark contrast to the more severe morbidity and mortality rates observed in other world regions (2).

Several factors contribute to the under-estimation and under-reporting of COVID-19 cases in Africa. The presence of asymptomatic infections, capable of transmitting the virus (5, 6), and a tendency of fewer clinical symptoms among the younger demographic (7), potentially lead to a significant under-estimation of the true case count, especially in regions with younger populations like Africa. Moreover, limited testing and public health resources, inadequate public awareness, cultural stigmatization, self-medication practices, and initially unestablished monitoring practices on the continent might have further contributed to the under-reporting of cases (8). Similarly, in some nations, political motives might influence data adjustment, impacting transparency, with certain governments altering their figures to project a particular narrative (8).

The WHO estimates that only one in seven cases was being detected in Africa (8), highlighting a substantial gap in data accuracy and completeness. Our previous investigation into the early phase of the COVID-19 outbreak across 54 African nations also revealed a significant under-reporting trend (9). Specifically, an average of only about 5.37% of all COVID-19 cases was duly reported. Strikingly, these numbers showed vast differences across nations, with Libya reporting a notably high rate of 30.41%, in contrast to São Tomé and Príncipe’s alarmingly low 0.02% (9).

These preceding observations highlight a pressing need to understand the underlying factors contributing to the reporting of COVID-19 cases in Africa. Our study aimed to explore the determinants influencing COVID-19 case detection and to classify African countries using these determinants. By identifying them, we aimed to provide insights that could be crucial for policymakers, health authorities, and lawmakers in crafting more adept public health strategies, refining resource allocation, and developing a more responsive and transparent system to confront the challenges presented by pandemics.

## 2 Methods

### 2.1 COVID-19 case reporting fractions

**Figure 1:**
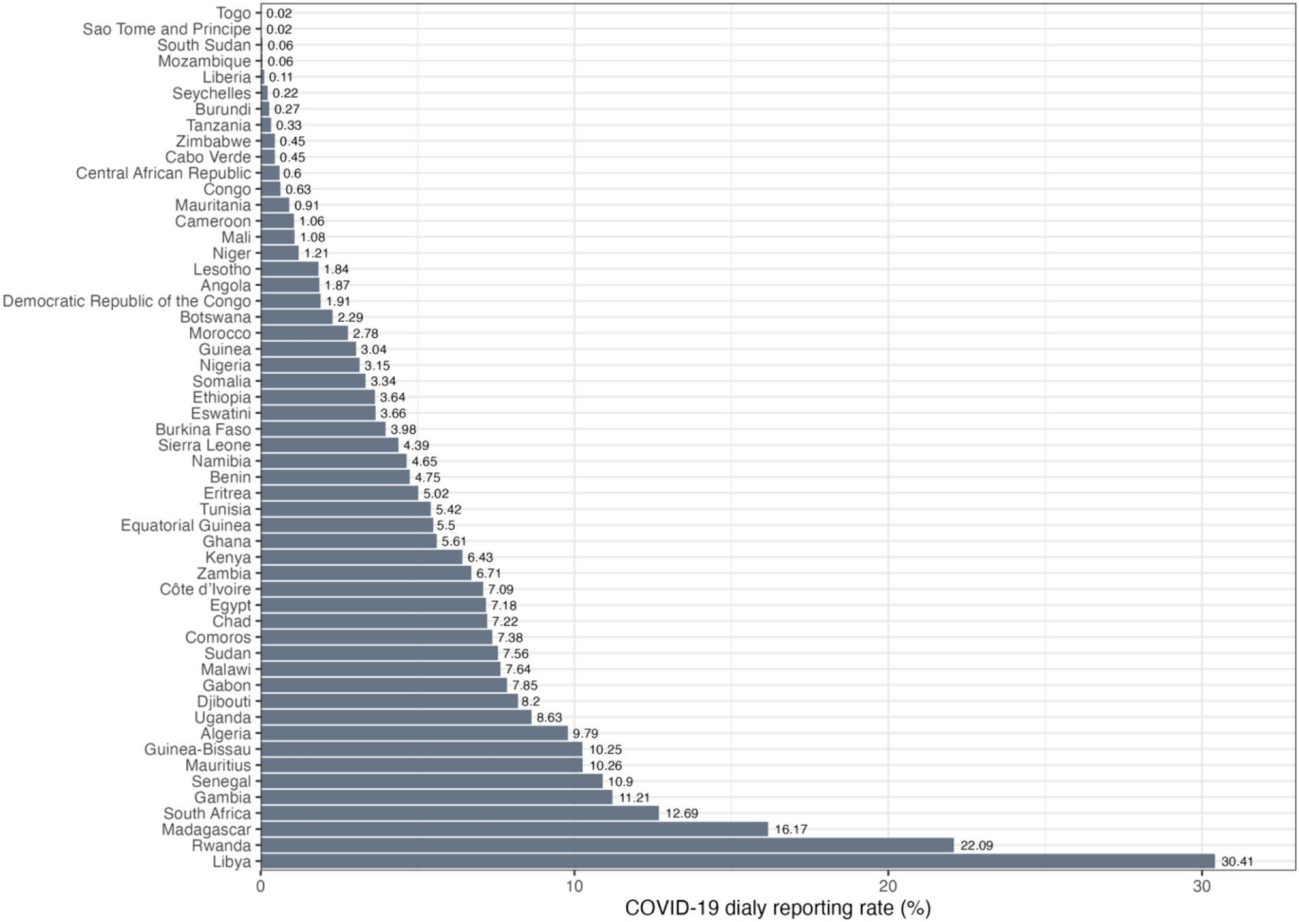
Daily COVID-19 reporting rates in percentage for 54 African countries in ascending order.

The fraction of overall COVID-19 cases reported was estimated for 54 African countries using a mathematical deterministic model with a Bayesian inference framework (9). In this study, we used these reporting rates as the response variable in the statistical analysis (Figure 1).

### 2.2 Factors

Our choice of variables was largely informed by past research and insights surrounding the under-reporting of COVID-19 cases in Africa. Factors like the occurrence of asymptomatic infections which are capable of virus transmission (5, 6), and a lower incidence of clinical symptoms among the younger demographic (7), have been pointed out, especially in regions with younger populations like Africa. Additionally, the constraints on testing and public health resources, lack of widespread public awareness, cultural stigma, self-medication practices, and initially unestablished monitoring practices on the continent are believed to have contributed to the under-reporting of cases (8). These considerations mirror challenges faced in other regions; for instance, in Brazil, demographic-related challenges led to misclassification of COVID-19 cases, particularly among younger individuals, those with lower education levels, and rβural residents (10). Such challenges highlighted the significant issue of misidentifying COVID-19 cases as severe acute respiratory infections (SARI). Education has also been pointed out as being critical for Africa’s health outcomes (11). Hence, we included the urban population, population literacy, female percentage, median age, percentage of Christians, and percentage of Muslims for each country to capture key demographic nuances.

Studies suggested variations in reported data due to a nation’s wealth, political climate, and inequalities (12). Notably, authoritarian governments were shown to skew data to appear competent and boost global reputation. Bureaucracies also modify data around elections to align with leadership’s wishes (13). Furthermore, democracy-related indicators, like the democracy index and world press freedom index, suggest countries with lower ranks, such as Turkey, China, Indonesia, and Iran, face greater COVID-19 under-reporting issues (14). To this end, for factors we also examined GINI index, GDP *per capita*, human development index, democracy index, press freedom index, political stability, government social media censorship, voice given to and accountability for citizens, corruption, ease of doing business, internet filtering, and air passenger volume.

Health-related factors, highlighted for their potential impact on reporting, were inspired from the results of study (15), which states that the number of tests conducted, global health security index, and average body mass index significantly correlated with reported COVID-19 cases per million population. These encompassed the number of nurses, number of physicians, public health expenditure in GDP, public health expenditure in total expenditure, body mass index, prevalence of cardiovascular diseases, prevalence of diabetes, global health security, cancer, prevalence of cholesterol, prevalence of lower respiratory infections, and malaria incidence.

All factors considered in this study are summarized in Table 1.

**Table 1:** List of factors considered with descriptions and sources.

Category	Variable	Year	Source
Demographics	Sex (female % in total population)	2020^*^	(16)
	Urban population (% of total population)	2020^*^	(17)
	Total population	2020	(18)
	Median age	2020	(19)
	Life expectancy	2019	(20)
	Population aged 65+ (% of total population)	2020	(21)
Socioeconomic	GINI coefficient (score from 0 to 100)	2019	(22)
	GDP *per capita* (gross domestic product divided by midyear population)	2020	(23)
	Democracy index (score from 0 to 10)	2020	(24)
	Human development index (score from 0 to 1)	2019	(25)
	Business: ease of doing business rank.	2019	(26)
	Air transport: passengers carried in thousands.	2019	(27)
	Social media: government social media censorship with score from 0 to 4 (4 = lowest/no censorship).	2020	(28)
	Internet: Government internet filtering with score from 0 to 4 (4 = lowest/no filtering).	2020	(28)
	Corruption perceptions index: perceived levels of public sector corruption, according to experts and business people.	2019	(29)
	Political stability and absence of violence or terrorism (standard normal distribution from approx.−2.5–2.5).	2019	(30)
	Voice and accountability: extent to which a country’s citizens are able to participate in selecting their government, as well as freedom of expression, freedom of association, and a free media (standard normal distribution from approx.−2.5–2.5).	2019	(30)
	Statistical capacity: overall country-level statistical capacity indicator (from 0 to 100, value of 100 indicating best capacity)	2019	(31)
	Press freedom score: measure of violence against journalists (from 0 to 100).	2019	(32)
Religion	Christianity (% of total population)	2010	(33)
	Islam (% of total population)	2020	(34, 35)
Education	Literacy rates: share of the population older than 14 years that is able to read and write.	2015^†^	(36)
Public health	Public health expenditure share of GDP (% of GDP)	2019	(37)
	Public health expenditure (% of total healthcare expenditure)	2019	(38)
	Cardiovascular diseases: death rate due to cardiovascular diseases (deaths per 100,000 population, both sexes, age-standardized)	2019	(39)
	Diabetes prevalence (% of population ages 20 to 79)	2017	(40)
	Lower respiratory infections rate (infections per 100,000 population)	2019	(41)
	Nurses: number of nurses and midwives per 1,000 population	2020	(42)
	Physicians: number of physicians per 1,000 population	2020	(43)
	BMI: mean body mass index of adults (in Kg/m^2^)	2016	(44)
	Global health security index (scale from 0–100)	2019	(45)
	Cancer: number of cases of neoplasms per 100 people in both sexes (%, age-standardized)	2019	(46)
	Malaria: number of new cases of malaria per 100,000 people, in both sexes	2019	(47)
	Cholesterol: mean Total Cholesterol (age-standardized)	2018	(48)

^*^Except for Eritrea, of which data is from 2011. ^†^Except for Djibouti and Somalia, of which data is from 2003 and 2001.

### 2.3 Statistical analysis

There are five missing values for malaria incidence (Lesotho, Libya, Mauritius, Seychelles, and Tunisia), three for GINI index (Equatorial Guinea, Eritrea, and Libya), two for GDP (Eritrea and South Sudan), and one for air transport (Mali), public health expenditure in GDP (Somalia), public health expenditure in total (Somalia), human development index and press freedom score (São Tomé and Príncipe). These missing values were imputed with mean values of corresponding variables in order to keep the original variable distributions. The numerical variables were then standardized to enable direct effect comparison.

To select the covariates to be used for the generalized additive model (GAM), we identified pairs of covariates that have a Spearman correlation greater than 0.6 or less than−0.6. For one covariate that has Spearman correlation above/below the threshold with more than one other covariates, we checked correlation between them and the response variable: if most other independent variables correlate higher with the dependent variable, this covariate will be dropped. Through this process, 12 factors were identified to be included in the drop list: population aged 65+, literacy, GINI index, press freedom, public health expenditure in GDP, public health expenditure in total, business, number of nurses, BMI, cancer prevalence, internet filtering and Christian population.

We used a GAM for the effects of the covariates on the COVID-19 daily case reporting rates. The general form of the model is


$$g\left(y_{i}\right)=\beta +\overset{n}{\underset{j=1}{\sum }}f_{j}\left(x_{ji}\right)+\epsilon_{i}\text{,}$$

where $y_{i}$ is the response variable, $x_{ji}\left(j=1,2,\ldots ,n\right)$ are the predictors, $\epsilon_{i}$ is identically and independently distributed as a normal random variable, g is a monotonous link function and $f_{j}\;\left(j=1,2,\ldots ,n\right)$ are nonparametric smoothing functions. Here we adopted cubic spline functions for continuous covariates and linear functions for categorical covariates. Compared to the data size, the GAM model has room for three more additional predictors after applying the drop list of aforementioned 12 factors. To get the best GAM, we added one predictor from the drop list at a time back to the GAM and kept the one that yields the largest deviance explained. Three rounds were run and internet filtering, number of nurses and literacy were added back to GAM after each round of selection.

### 2.4 Hierarchical clustering of principal components analysis

Using the COVID-19 daily reporting rates and the significant continuous factors from the statistical analysis, we clustered the 54 African countries through Hierarchical Clustering of Principal Components Analysis (HCPC) performed by packages “FactoMineR” and “factoextra” in RStudio2023.06.1 + 524. The algorithm is done in two major steps: first it reduces the dimension of the data through principal component analysis (PCA), and second cluster analysis is conducted on the PCA results using the Ward’s criterion which minimizes the total within-cluster variance.

## 3 Results

**Figure 2:**
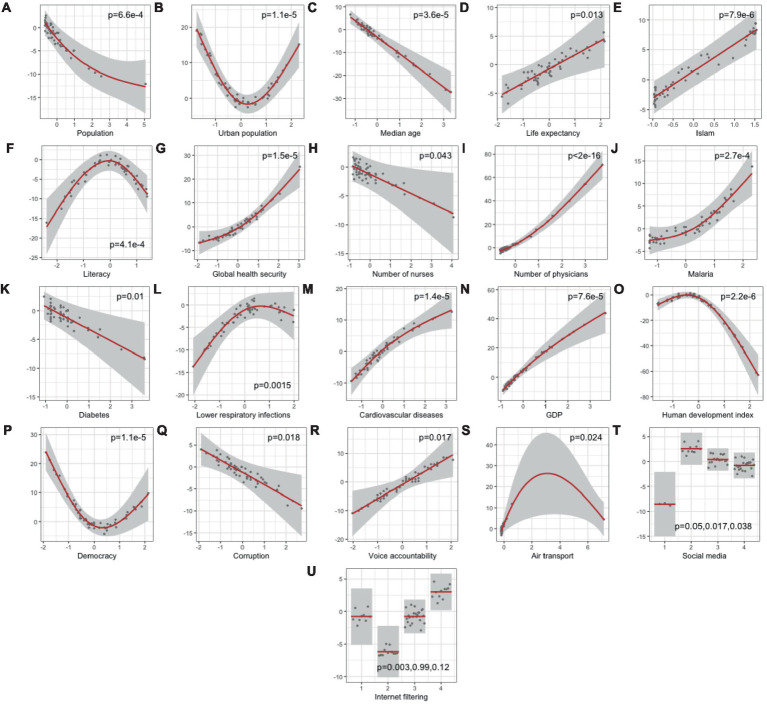
Partial effects of statistically significant predictors **(A–U)** on COVID-19 daily case reporting rate (%) with *p*-values from generalized additive model (Deviance explained = 97.4%). *p*-values<0.05 were considered statistically significant.

The deviance explained for the best GAM model with identity link function is 97.4% (adjusted R-squared: 0.897), indicating a high explanatory power from the model. Among the 34 covariates, the significant factors with p -values less than 0.05 are: population, urban population, median age, life expectancy, Islam population, literacy rates, global health security, number of nurses, number of physicians, malaria incidence, diabetes prevalence, lower respiratory infections prevalence, cardiovascular diseases prevalence, GDP, human development index, democracy index, corruption index, voice accountability, air transport, all levels of social media and level two of internet filtering. Their partial effects on the COVID-19 daily case reporting rates are shown in Figure 2. As in Figure 2, life expectancy, Islam population, global health security, number of physicians, malaria, cardiovascular diseases prevalence, GDP and voice accountability are positively correlated with higher COVID-19 reporting, while total population, median age, number of nurses, diabetes prevalence and corruption index are negatively correlated with higher COVID-19 reporting. Intermediate levels of urban population and democracy index correlate with lower reporting rates, while intermediate levels of literary rates, lower respiratory infections prevalence, human development index and air transport correlate with higher reporting rates.

**Figure 3:**
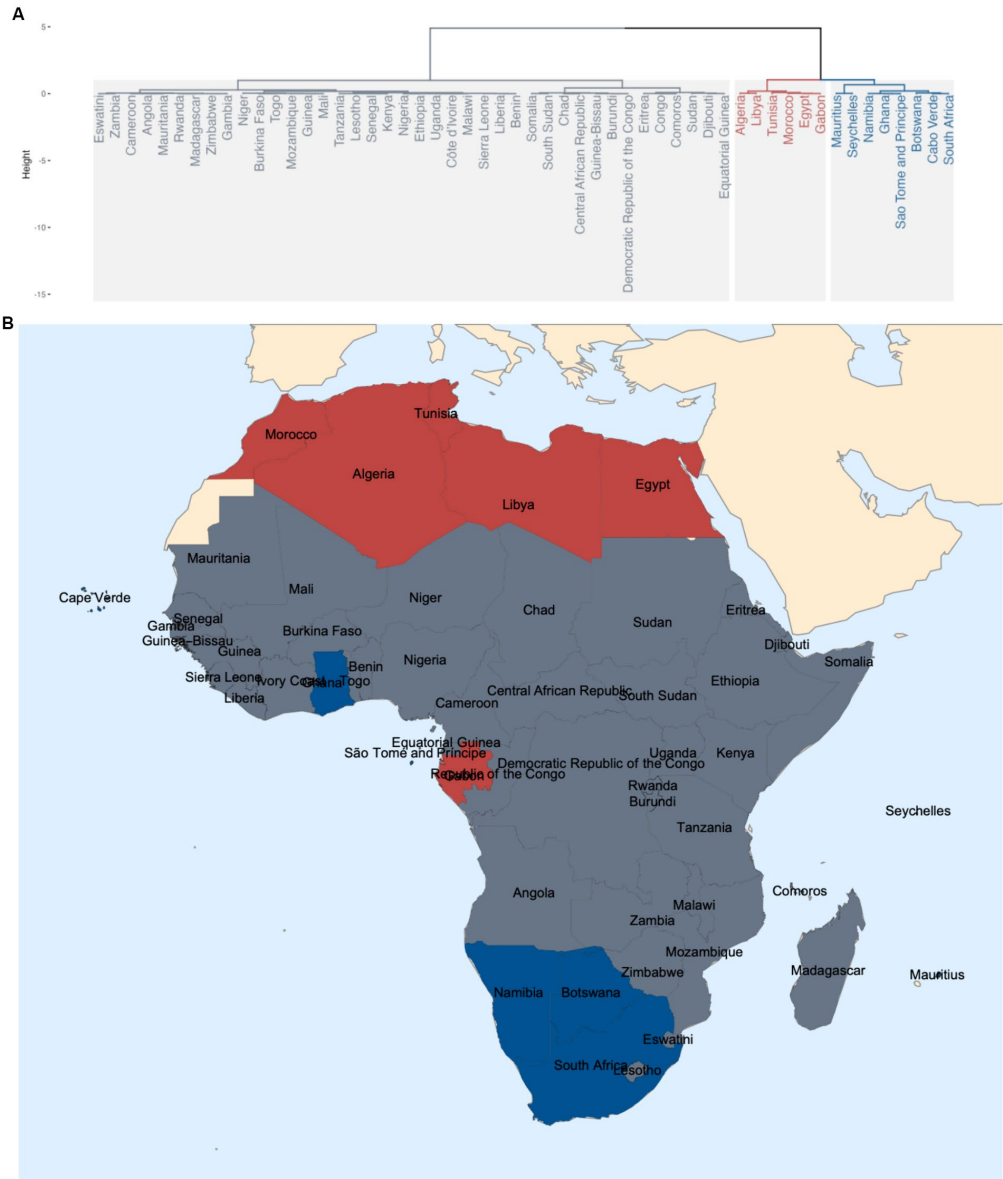
**(A)** Dendrogram showing three major clusters from HCPC; **(B)** Geographical locations of the three clusters.

HCPC analysis resulted in three major clusters as shown in the dendrogram in Figure 3A, indicated by different colors. The mean Silhouette score for the clustering using the coordinates in the factor map is 0.485. Their geographical locations are shown in Figure 3B with corresponding colors for each resultant cluster. Basically, Cluster 1 (gray) includes the central African region with the largest number of countries. Cluster 2 (red) includes the northern African region with Morocco, Tunisia, Alegria, Libya and Egypt (Gabon as an exception). Cluster 3 (blue) includes essentially the southern African region with Namibia, Botswana, South Africa (Ghana as an exception), plus Mauritius, Seychelles, Cabo Verde and São Tomé and Príncipe.

**Figure 4:**
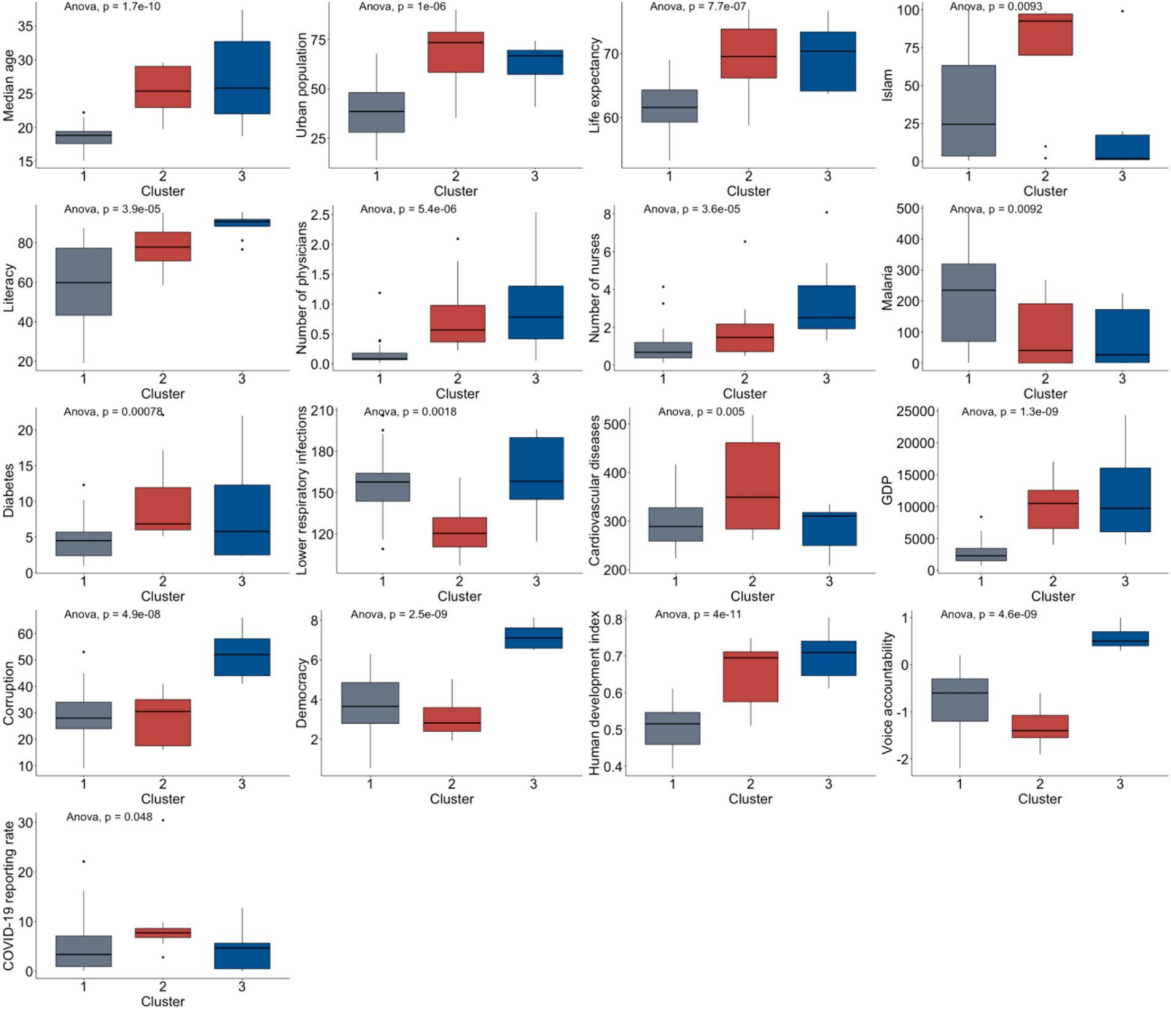
Significant covariates characterizing the three major clusters from HCPC.

Among all continuous covariates and the COVID-19 daily case reporting rate used for the clustering, the reporting rate ( p=0.048 ), median age ( $p=1.7\times 10^{-10}$ ), urban population ( $p=1\times 10^{-6}$ ), life expectancy ( $p=7.7\times 10^{-7}$ ), Islam population ( p=0.0093 ), literacy rate ( $p=3.9\times 10^{-5}$ ), number of physicians ( $p=5.4\times 10^{-6}$ ), number of nurses ( $p=3.6\times 10^{-5}$ ), malaria incidence ( p=0.0092 ), diabetes prevalence ( p=0.00078 ), lower respiratory infections prevalence ( p=0.0018 ), cardiovascular diseases prevalence ( p=0.005 ), GDP ( $p=1.3\times 10^{-9}$ ), corruption index ( $p=4.9\times 10^{-8}$ ), democracy index ( $p=2.5\times 10^{-9}$ ), human development index ( $p=4\times 10^{-11}$ ) and voice accountability ( $p=4.6\times 10^{-9}$ ) are significantly different across clusters from the ANOVA test, as is shown in Figure 4. According to Figure 4, Cluster 2 (red) has the best COVID-19 reporting performance, followed by Cluster 3 (blue) and then Cluster 1 (gray). Among the three major clusters, Cluster 2 and 3 on average exhibit higher median age, life expectancy, urban population size, human development index, GDP, number of physicians, number of nurses and literacy rates and lower malaria incidence. However, Cluster 2 and 3 on average also have higher corruption index, diabetes and cardiovascular diseases prevalence. Cluster 1 shows intermediate average values with Cluster 2 and 3 polarized in democracy index, voice accountability, lower respiratory infections prevalence and Islamic population size.

## 4 Discussion

We identified four demographic factors: total population, urban population, median age and life expectancy; seven public health related factors: global health security, number of physicians, number of nurses, malaria, diabetes, lower respiratory diseases and cardiovascular diseases prevalence; eight socioeconomic factors: GDP, human development index, democracy index, corruption index, voice accountability, air transport, social media and internet filtering; one religious factor Islamic population and one education factor literacy rates as significantly associated with COVID-19 case identification during the early stage of the pandemic in Africa. Based on these determinants and the estimated daily case reporting rate, the African countries can be essentially categorized into three major clusters: northern, southern and central, where the northern region has the highest COVID-19 case detection, followed by the southern region, while the central region has the poorest performance in case detection.

Beginning with demographics, we note that areas with a low urban population might see more contained and easier-to-track spread, while highly urbanized areas could boast better health infrastructure and reporting systems (Figure 2B). Middle urban areas may face reporting challenges as rapidly growing populations outpace healthcare facilities’ development (49). Countries with a higher median age (Figure 2C) might experience a higher COVID-19 case fatality rate due to increased risk in older individuals (7, 50), significantly straining healthcare systems. Higher life expectancy (Figure 2D) could indicate superior overall healthcare systems (51, 52), potentially leading to more efficient COVID-19 detection and reporting. The negative association with population size (Figure 2A) (53) suggests that larger populations face lower reporting rates due to the challenges of scaling up testing and reporting infrastructure (54, 55) or the greater likelihood of under-detection in densely populated areas.

Socioeconomically, a higher GDP (Figure 2N) might correlate with better healthcare infrastructure (56, 57), thus enhancing testing and reporting capacity. The democracy index (Figure 2P) suggests that countries with very high (58) or very low (59) scores exhibit higher reporting rates, possibly reflecting transparency in democratic countries and international scrutiny or aid in less democratic countries (60). In general, during the COVID-19 pandemic, many countries experienced declines in their democracy scores, particularly those with authoritarian regimes (61, 62). Increased corruption (Figure 2Q) is linked to decreased reporting rates, suggesting that corruption may hinder accurate reporting (63) due to factors like mismanagement of information and resources. Countries where citizens have more voice and accountability (Figure 2R) may report better, likely due to greater transparency and public demand for accurate information. This could also be seen in social media censorship (Figure 2T), but beyond a certain level, the improvement on case reporting becomes less pronounced. The sharp decline at the lower end of the health development index (HDI) (Figure 2O) scale might suggest that countries with lower development levels have much lower COVID-19 reporting rates, possibly due to limited healthcare infrastructure and fewer resources for testing and reporting. As countries attain a moderate level of human development, the reporting rate’s decrease slows, potentially indicating that beyond a certain threshold of development, improvements in reporting rates become less pronounced, possibly due to the establishment of basic reporting systems. Countries with moderate levels of air transport have the highest reporting rates (Figure 2S), which could be tied to better connectivity and infrastructure that also supports health reporting systems.

Interestingly, a higher proportion of the Muslim population (Figure 2E) correlates with increased reporting rates, prompting consideration of the complex interplay between religious practices, community engagement, and public health policies. This finding warrants further investigation to understand the underlying social mechanisms at play. The inverse U-shaped curve observed for literacy rates (Figure 2F) suggests that moderate literacy aligns with the highest reporting rates, likely due to the intersection of disease prevalence and public health awareness. Countries with high literacy rates might have lower incidence due to preventative measures and thus fewer under-reporting, while low literacy might impede disease recognition and reporting.

Public health insights indicate varying impacts of healthcare resources on COVID-19 reporting rates. Higher malaria incidence (Figure 2J) (64) may indicate robust disease tracking systems (65). Interestingly, the number of nurses *per capita* (Figure 2H) has a negative association with reporting rates, suggesting more nurses might not directly translate to higher reporting, potentially due to efficient disease management or other unaccounted factors. Conversely, a higher number of physicians (Figure 2I) correlates with increased reporting rates, possibly indicating better diagnostic and surveillance capacity. It could also reflect broader healthcare system quality, where more physicians *per capita* mean more comprehensive care, including chronic disease management, and a heightened awareness and ability to report infectious diseases like COVID-19. The global health security index’s positive association (Figure 2G) implies that countries prepared for health crises report more efficiently (66). This result would imply that countries with higher scores in global health security, which encompasses factors like disease detection, response, health system quality, and the risk environment, are likely to report COVID-19 cases more efficiently and accurately. This is consistent with what would be expected, as a higher global health security index indicates a stronger healthcare infrastructure capable of dealing with pandemics. For diabetes prevalence (Figure 2K), a negative association would suggest that as the prevalence of diabetes in the population increases, the COVID-19 reporting rates actually decrease. This could be due to several potential factors. For example, countries with higher prevalence of diabetes may have healthcare systems that are more burdened by chronic disease management (67), possibly leading to less capacity for effective infectious disease surveillance and reporting. Alternatively, it could reflect socio-economic factors (68), where high diabetes prevalence is associated with other factors that might impede effective reporting, such as limited access to healthcare services or lower health literacy regarding infectious diseases. The inverse U-shaped relationship between lower respiratory infection (LRI) rates and COVID-19 reporting (Figure 2L) suggests that countries with extremely high or low LRI rates tend to have lower COVID-19 reporting rates, while those with moderate LRI rates report more cases. High LRI rates may strain health resources, leading to under-reporting of COVID-19, whereas countries with low LRI rates might lack the necessary infrastructure or experience to detect and report COVID-19 effectively. Optimal reporting is observed in countries with moderate LRI prevalence, possibly due to balanced health system vigilance and capacity. Notable exceptions, such as Libya with low LRI but high COVID-19 reporting rates, indicate that other factors also significantly influence reporting. The positive association seen with cardiovascular disease death rates (Figure 2M) suggests that countries with a greater number of reported deaths from cardiovascular diseases have a greater COVID-19 reporting rate. As cardiovascular related diseases are the leading cause of death in Africa (69), countries with a higher number of reported deaths from such diseases would also possess enhanced disease surveillance and reporting practices and thus have a higher COVID-19 reporting rate.

Studies have been conducted to find possible reasons for the low numbers of cases and deaths in Africa. The proportion of older adult people ( ≥ 60 years old) was identified to be the major factor to explain low case number, and the health systems capacities was identified to be responsible for the case under-estimation in one study (70). In another study, international flights, testing capacity, population density, young population, Vitamin D levels, cross-immunity from other infections, temperature and UV light and humidity were listed as potential reasons for Africa’s low case number (71). However, it is worth note that our study is different as the subject of this study is the reporting/under-reporting rate, rather than the overall case number. To our knowledge, there have not been investigations on the determinants of COVID-19 case reporting in Africa. The dependent variable of COVID-19 daily case reporting fraction was estimated using the same mechanistic mathematical model for all African countries and therefore provide a reliable and fair comparison among them (9). Our study also considered a wider range of potential factors than those in existing literature (10, 12–15). Interestingly, some factors do not show the same relationship with total case number and case reporting ratio. For example, higher air transport rate and human development index are contemplated to associate with higher COVID-19 case number (71), but they demonstrate an inverse U-shaped rather than monotonic association with the reporting ratio (Figure 2). And while larger median age implies more cases, its relationship with reporting ratio is negative (Figure 2D). That could provoke further thinking on how the factors affect the case reporting system. The clustering results from our HCPC analysis are in general agreement with other studies. For example, previous emerging infectious diseases epidemics revealed the vulnerability of Western and Central Africa in facing both known and unknown pathogens due to a growing urban population with insufficient public health infrastructure (72), and moreover, Northern and Southern Africa show higher capacities in health systems (70). However, it should be noted that reporting practice varies as the outbreak progresses, and the case reporting rate used here is only the estimate for the initial phase of the pandemic. Therefore, these determinants could be interpretated as the preparedness in face of a novel emerging communicable disease outbreak but cannot be extended to subsequent efforts made by the nations or the overall case identification performance over the entire course.

## Data availability statement

Publicly available datasets were analyzed in this study. This data can be found here: all datasets for this study can be found through the links in the corresponding references.

## Author contributions

QH: Conceptualization, Methodology, Software, Visualization, Writing – original draft, Data curation, Formal analysis. GR: Data curation, Formal analysis, Software, Writing – original draft. MM: Methodology, Writing – original draft. WA: Conceptualization, Data curation, Writing – original draft. AAh: Supervision, Writing – review & editing. AAs: Supervision, Writing – review & editing. JO: Supervision, Writing – review & editing. JW: Supervision, Writing – review & editing. JK: Conceptualization, Funding acquisition, Supervision, Writing – review & editing.

## References

[1] Andersen KG Rambaut A Lipkin WI Holmes EC Garry RF. The proximal origin of sars-cov-2. Nat. medicine. (2020) 26:450–2. doi: 10.1038/s41591-020-0820-9, PMID: 32284615 PMC7095063

[2] WHO. Who coronavirus (covid-19) dashboard. (2022). Available at: https://covid19.who.int

[3] Africa news. Covid-19: Egypt confirms first coronavirus case in africa. Coronavirus outbreak news. (2020). Available at: https://www.aljazeera.com/news/2020/02/egypt-confirms-coronavirus-case-africa-200214190840134.html

[4] WHO. Coronavirus (covid-19): WHO African region numbers at a glance. (2022). Avaiable at:https://www.afro.who.int/health-topics/coronavirus-covid-19

[5] Bai Y Yao L Wei T Tian F Jin DY Chen L. Presumed asymptomatic carrier transmission of covid-19. J Am Med Assoc. (2020) 323:1406–7. doi: 10.1001/jama.2020.2565, PMID: 32083643 PMC7042844

[6] Rivett L Sridhar S Sparkes D Routledge M Jones NK. Screening of healthcare workers for sars-cov-2 highlights the role of asymptomatic carriage in covid-19 transmission. Elife. (2020) 9:e58728. doi: 10.7554/eLife.58728 32392129 PMC7314537

[7] Davies NG Klepac P Liu Y Prem K Eggo RM. Age-dependent effects in the transmission and control ofcovid-19 epidemics. Nat medicine. (2020) 26:1–7. doi: 10.1038/s41591-020-0962-9 32546824

[8] WHO. Six in seven covid-19 infections go undetected in Africa. (2021). Available at: https://www.afro.who.int/news/six-seven-covid-19-infections-go-undetected-africa

[9] Han Q Bragazzi N Asgary A Orbinski J Wu J Kong JD. Estimation of epidemiological parameters and ascertainment rate from early transmission of COVID-19 across Africa. R Soc Open Sci. (2023) 10:230316. doi: 10.1098/rsos.230316, PMID: 37736525 PMC10509578

[10] Sansone NMS Boschiero MN Marson FAL. Epidemiologic profile of severe acute respiratory infection in Brazil during the COVID-19 pandemic: an epidemiological study. Front Microbiol. (2022) 13:911036. doi: 10.3389/fmicb.2022.911036, PMID: 35854935 PMC9288583

[11] Azevedo M. J. Azevedo M. J. (2017). The state of health system (s) in Africa: challenges and opportunities. In: Azevedo MJ, editor. Historical Perspectives on the State of Health and Health Systems in Africa, Volume II: The Modern Era. Cham: Springer International Publishing, 1–73

[12] Landman T Smallman-Raynor M. The politics of COVID-19: Government response in comparative perspective. Politic Geograp. (2023) 106:102957. doi: 10.1016/j.polgeo.2023.102957

[13] Kofanov D Kozlov V Libman A Zakharov N. Encouraged to cheat? Federal Incentives and career concerns at the sub-national level as determinants of under-reporting of COVID-19 mortality in Russia. Br J Polit Sci. (2023) 53:835–60. doi: 10.1017/S0007123422000527

[14] Kisa S Kisa A. Under-reporting of Covid-19 cases in Turkey. Int J Health Plann Manag. (2020) 35:1009–13. doi: 10.1002/hpm.3031, PMID: 32744745 PMC7436880

[15] Bouba Y Tsinda EK Fonkou MDM Mmbando GS Bragazzi NL Kong JD. The determinants of the low COVID-19 transmission and mortality rates in Africa: a cross-country analysis. Front Public Health. (2021) 9:751197. doi: 10.3389/fpubh.2021.751197, PMID: 34746085 PMC8568130

[16] The World Bank. Population, female (% of total) | data. (2021). Available at: https://data.worldbank.org/indicator/SP.POP.TOTL.FE.ZS

[17] The World Bank. Urban population (% of total) | data. (2021). Available at: https://data.worldbank.org/indicator/SP.URB.TOTL.IN.ZS

[18] United Nations. World population prospects-population division-United Nations. (2022). Available at: https://population.un.org/wpp/Download/Standard/Population/

[19] WHO. Indicator Metadata Registry Details. Available at: https://www.who.int/data/gho/indicator-metadata-registry/imr-details/116#:~:text=Definiti

[20] The World Bank. Life expectancy at birth, total (years). (2020). Available at: https://data.worldbank.org/indicator/SP.DYN.LE00.IN

[21] UN, World Population Prospects (2022) – 2022–Processed by our world in data. “Ages 65+” [dataset]. UN, world population prospects. Available at: https://ourworldindata.org/age-structure

[22] The World Bank. GINI index (World Bank estimate). (2021). Available at: https://data.worldbank.org/indicator/SI.POV.GINI

[23] World Bank. GDP per capita (current US$). (2021). Available at: https://data.worldbank.org/indicator/NY.GDP.PCAP.CD

[24] Economist intelligence unit. Democracy Index 2021. (2021). Available at: https://www.eiu.com/n/campaigns/democracy-index-2020/10.1093/inthealth/ihu064 25280474

[25] Roser M. Human Development Index (HDI). (2019). Available at: https://ourworldindata.org/human-development-index

[26] World Bank. Ease of doing business index (1=most business-friendly regulations). (2021). Available at: https://data.worldbank.org/indicator/IC.BUS.EASE.XQ?end=2019&start=2019&view=bar

[27] The World Bank. Air transport, passengers carried. (2019). Available at: https://data.worldbank.org/indicator/is.air.psgr

[28] Digital Society Project. (2024). Available at: http://digitalsocietyproject.org/

[29] Transparency International. 2019- CPI. (2019). Available at: https://www.transparency.org/en/cpi/2019

[30] The World Bank. Worldwide governance indicators. (2021). Available at: https://databank.worldbank.org/source/worldwide-governance-indicators

[31] The World Bank, Statistical Capacity Indicators. (2024). Available at: https://databank.worldbank.org/source/statistical-capacity-indicators

[32] Press freedom index. Our world in data. (2022). Available at: https://ourworldindata.org/grapher/press-freedom-index-rsf

[33] Author N. Global Christianity - a report on the size and distribution of the World’s Christian population. (2011). Available at: https://www.pewresearch.org/religion/2011/12/19/global-christianity-exec/#global-distribution-of-christians

[34] United States Department of State. 2022 Report on International Religious Freedom-United States Department of State. (2023). Available at: https://www.state.gov/reports/2022-report-on-international-religious-freedom/

[35] Central Intelligence Agency. Religions-the world Factbook. Available at: https://www.cia.gov/the-world-factbook/field/religions/

[36] Literacy rate. Our world in data. (2023). Available at: https://ourworldindata.org/grapher/cross-country-literacy-rates?tab=map&country=East+Asia+%26+Pacific~Sub-Saharan+Africa~Europe+%26+Central+Asia~Latin+America+%26+Caribbean~Middle+East+%26+North+Africa~South+Asia

[37] Public healthcare expenditure as a share of GDP. Our world in data. (2023). Available at: https://ourworldindata.org/grapher/public-healthcare-spending-share-gdp

[38] Our world in data. Public expenditure on healthcare as percent of total healthcare expenditure. (2023). Available at: https://ourworldindata.org/grapher/share-of-public-expenditure-on-healthcare-by-country?tab=table

[39] Our world in data. Death rate from cardiovascular disease. (2019). Available at: https://ourworldindata.org/grapher/cardiovascular-disease-death-rates

[40] World Bank. Diabetes prevalence (% of population ages 20 to 79). (2017). Available at: https://data.worldbank.org/indicator/SH.STA.DIAB.ZS

[41] Institute for Health Metrics and Evaluation. GBD Results. (2019). Available at: https://vizhub.healthdata.org/gbd-results/

[42] World Bank Open Data. Nurses and midwives (per 1,000 people). (2020). Available at: https://data.worldbank.org/indicator/SH.MED.NUMW.P3?end=2018&name_desc=false&start=2018&view=chart

[43] World Bank Open Data. Physicians (per 1,000 people). (2020). Available at: https://data.worldbank.org/indicator/SH.MED.PHYS.ZS?end=2018&name_desc=false&start=2018&view=chart

[44] WHO. Mean BMI (kg/m2) (age-standardized estimate). (2017). Available at: https://www.who.int/data/gho/data/indicators/indicator-details/GHO/mean-bmi-(kg-m-)-(age-standardized-estimate)

[45] GHS Index. The Global Health security Index; (2019). Available at: https://www.ghsindex.org/

[46] Our World in data. Share of population with cancer. (2024). Available at: https://ourworldindata.org/grapher/share-of-population-with-cancer?tab=table

[47] Our World in data. Incidence of malaria. (2023). Available at: https://ourworldindata.org/grapher/the-incidence-of-malaria-per-1000-population?tab=table

[48] Mean Total Cholesterol (age-standardized estimate). (2021). Available from: https://www.who.int/data/gho/data/indicators/indicator-details/GHO/mean-total-cholesterol-(age-standardized-estimate)

[49] Ruszczyk H Castán Broto V McFarlane C. Urban health challenges: lessons from COVID-19 responses. Geoforum. (2022) 131:105–15. doi: 10.1016/j.geoforum.2022.03.003 35291575 PMC8913332

[50] Wang H Paulson KR Pease SA Watson S Comfort H Zheng P. Estimating excess mortality due to the COVID-19 pandemic: a systematic analysis of COVID-19-related mortality, 2020–21. Lancet. (2022) 399:1513–36. doi: 10.1016/S0140-6736(21)02796-3 35279232 PMC8912932

[51] Galvani-Townsend S Martinez I Pandey A. Is life expectancy higher in countries and territories with publicly funded health care? Global analysis of health care access and the social determinants of health. Journal of. Glob Health. (2022) 12:12. doi: 10.7189/jogh.12.04091 PMC9653205 36370409

[52] OECD. What has driven life expectancy gains in recent decades? A cross-country analysis of OECD member states. Paris: OECD iLibrary (2017).

[53] Msemburi W Karlinsky A Knutson V Aleshin-Guendel S Chatterji S Wakefield J. The WHO estimates of excess mortality associated with the COVID-19 pandemic. Nature. (2022) 613:130–7. doi: 10.1038/s41586-022-05522-2 36517599 PMC9812776

[54] Barker PM Reid A Schall MW. A framework for scaling up health interventions: lessons from large-scale improvement initiatives in Africa. Implement Sci. (2015) 11:12. doi: 10.1186/s13012-016-0374-x PMC4731989 26821910

[55] Mangham LJ Hanson K. Scaling up in international health: what are the key issues? Health Policy Plan. (2010) 25:85–96. doi: 10.1093/heapol/czp066 20071454

[56] Bedir S. Healthcare expenditure and economic growth in developing countries. Adv Econ Bus. (2016) 4:76–86. doi: 10.13189/aeb.2016.040202

[57] Fedeli S. The impact of GDP on health care expenditure: the case of Italy (1982–2009). Soc Indic Res. (2014) 122:347–70. doi: 10.1007/s11205-014-0703-x

[58] Yao L Li M Wan JY Howard SC Bailey JE Graff JC. Democracy and case fatality rate of COVID-19 at early stage of pandemic: a multicountry study. Environ Sci Pollut Res. (2021) 29:8694–704. doi: 10.1007/s11356-021-16250-x PMC8421237 34490579

[59] Karabulut G Zimmermann KF Bilgin MH Doker AC. Democracy and COVID-19 outcomes. Econ Lett. (2021) 203:109840. doi: 10.1016/j.econlet.2021.109840, PMID: 33814654 PMC7997903

[60] Alesina A Dollar D. Who gives foreign aid to whom and why? J Econ Growth. (2000) 5:33–63. doi: 10.1023/A:1009874203400

[61] Economist Intelligence. Covid-19 pandemic causes a global democracy slump. (2021). Available at: https://www.eiu.com/n/covid-19-pandemic-causes-a-global-democracy-slump/

[62] Sorsa V-P Kivikoski K. COVID-19 and democracy: a scoping review. BMC Public Health. (2023) 23:1668. doi: 10.1186/s12889-023-16172-y, PMID: 37649016 PMC10469824

[63] Usman M Husnain M Akhtar MW Ali Y Riaz A Riaz A. From the COVID-19 pandemic to corrupt practices: a tale of two evils. Environ Sci Pollut Res. (2022) 29:30297–310. doi: 10.1007/s11356-022-18536-0, PMID: 35000178 PMC8742567

[64] Habibzadeh F. Malaria and the incidence of COVID-19 in Africa: an ecological study. BMC Infect Dis. (2023) 23:66. doi: 10.1186/s12879-023-08032-2, PMID: 36737728 PMC9896446

[65] Kerr G Robinson LJ Russell TL Macdonald J. Lessons for improved COVID-19 surveillance from the scale-up of malaria testing strategies. Malar J. (2022) 21:223. doi: 10.1186/s12936-022-04240-4 35858916 PMC9296766

[66] Ledesma JR Isaac CR Dowell SF Blazes DL Essix GV Budeski K. Evaluation of the Global Health Security Index as a predictor of COVID-19 excess mortality standardised for under-reporting and age structure. BMJ Glob. Health. (2023) 8:e012203. doi: 10.1136/bmjgh-2023-012203 PMC10335545 37414431

[67] Liu J Bai R Chai Z Cooper ME Zimmet PZ Zhang L. Low-and middle-income countries demonstrate rapid growth of type 2 diabetes: an analysis based on global burden of disease 1990–2019 data. Diabetologia. (2022) 65:1339–52. doi: 10.1007/s00125-022-05713-6 35587275 PMC9118183

[68] Richards SE Wijeweera C Wijeweera A. Lifestyle and socioeconomic determinants of diabetes: evidence from country-level data. PLOS ONE. (2022) 17:e0270476. doi: 10.1371/journal.pone.0270476 35901054 PMC9333224

[69] Doku A Tuglo LS Chilunga F Edzeame J Peters RJG Agyemang C. A multilevel and multicenter assessment of health care system capacity to manage cardiovascular diseases in Africa: a baseline study of the Ghana heart initiative. BMC. (2023) 23:421. doi: 10.1186/s12872-023-03430-5, PMID: 37620790 PMC10464459

[70] Thenon N Peyre M Huc M Touré A Roger F Mangiarotti S. COVID-19 in Africa: underreporting, demographic effect, chaotic dynamics, and mitigation strategy impact. PLoS Negl Trop Dis. (2022) 16:e0010735. doi: 10.1371/journal.pntd.0010735, PMID: 36112718 PMC9518880

[71] Bamgboye EL Omiye JA Afolaranmi OJ Davids MR Tannor EK Wadee S. COVID-19 pandemic: is Africa different? J Natl Med Assoc. (2021) 113:324–35. doi: 10.1016/j.jnma.2020.10.001, PMID: 33153755 PMC7607238

[72] Munster VJ Bausch DG De Wit E Fischer R Kobinger G Muñoz-Fontela C. Outbreaks in a rapidly changing Central Africa—lessons from Ebola. N Engl J Med. (2018) 379:1198–201. doi: 10.1056/NEJMp1807691, PMID: 30134126

